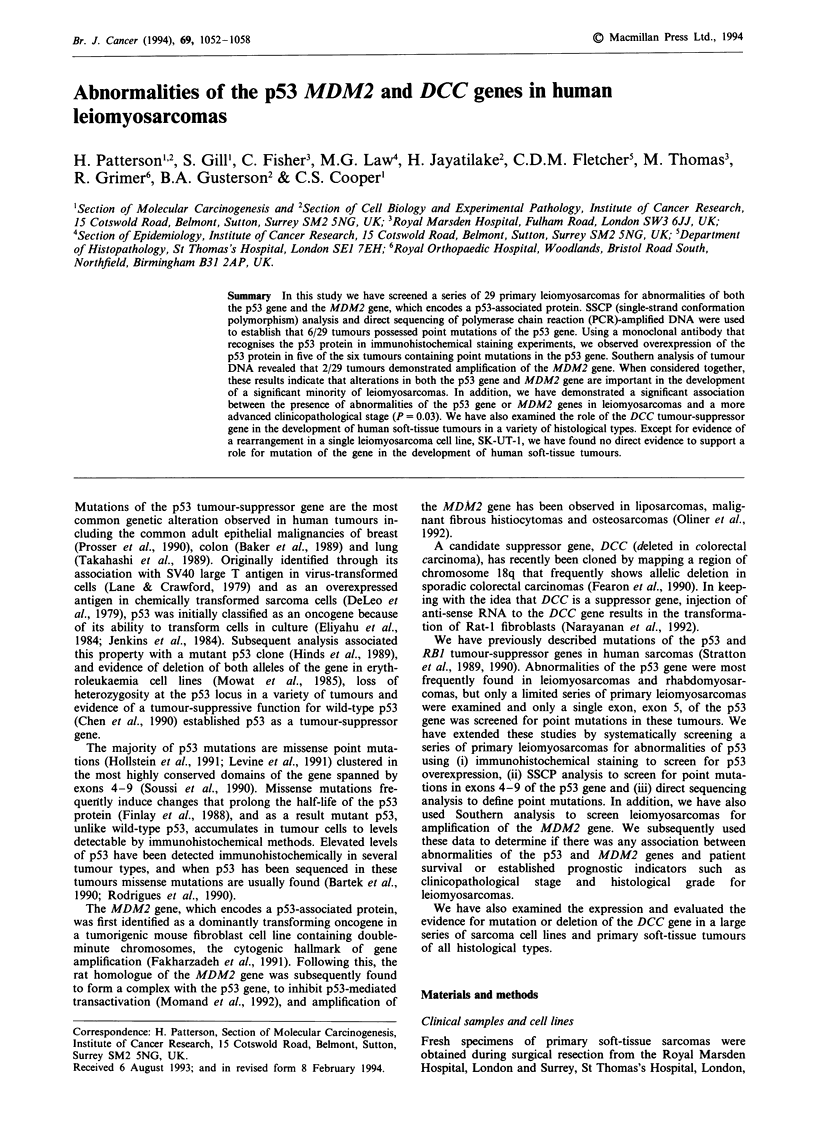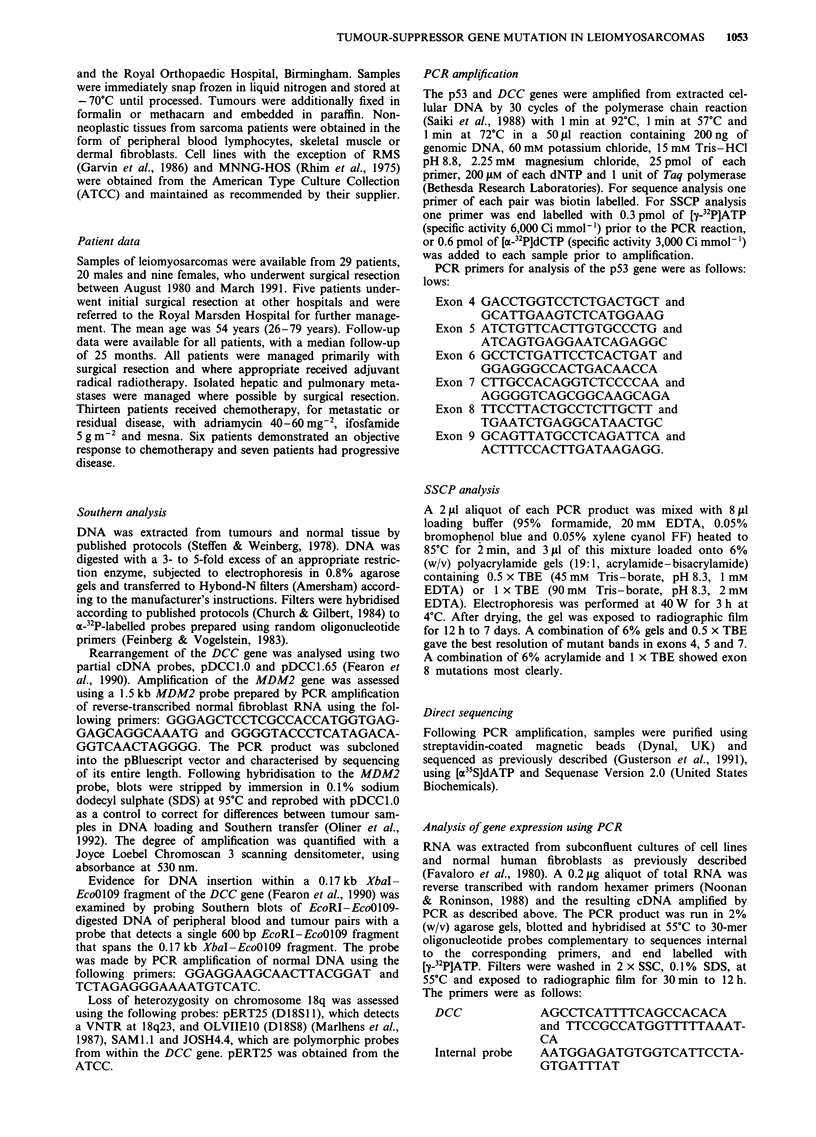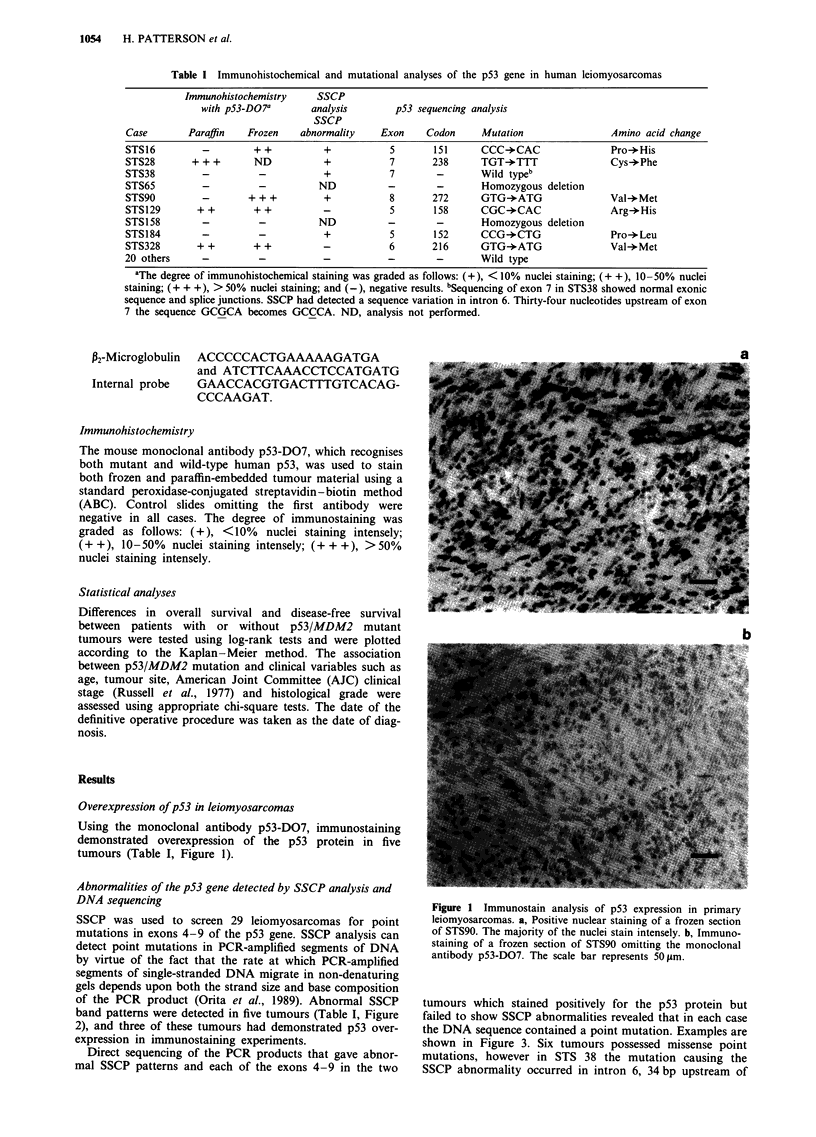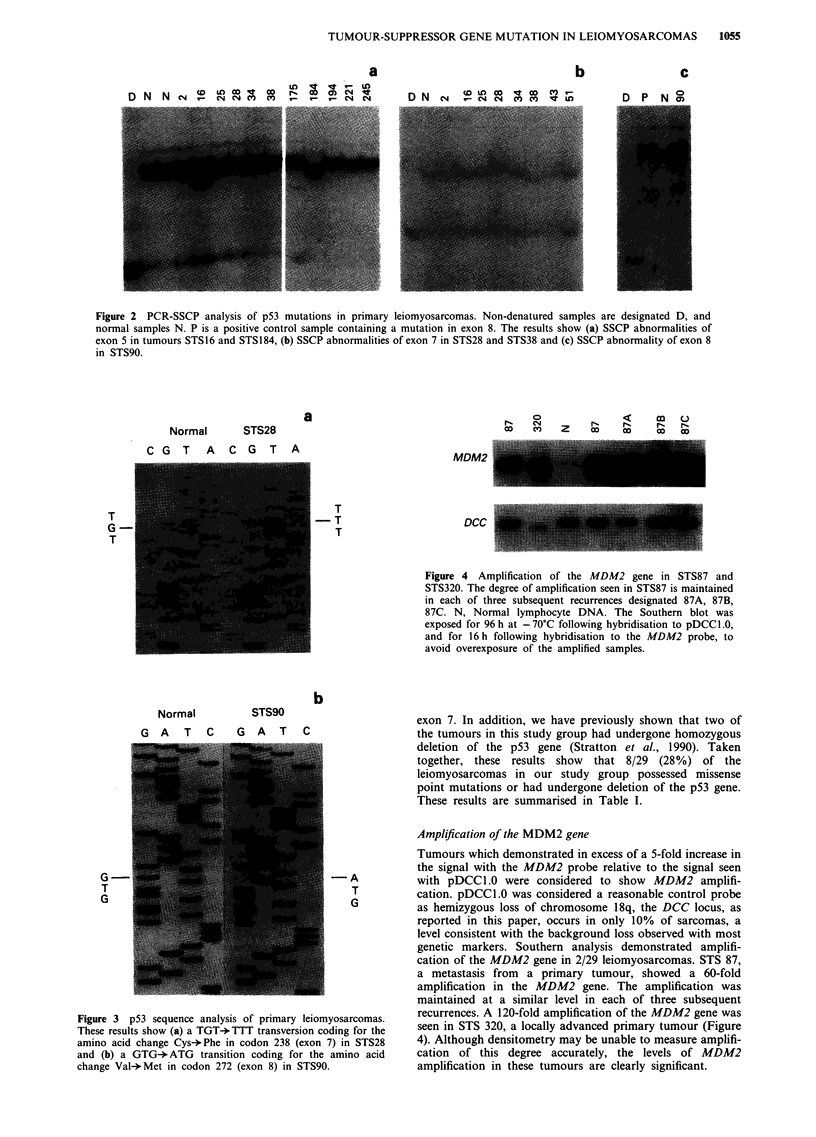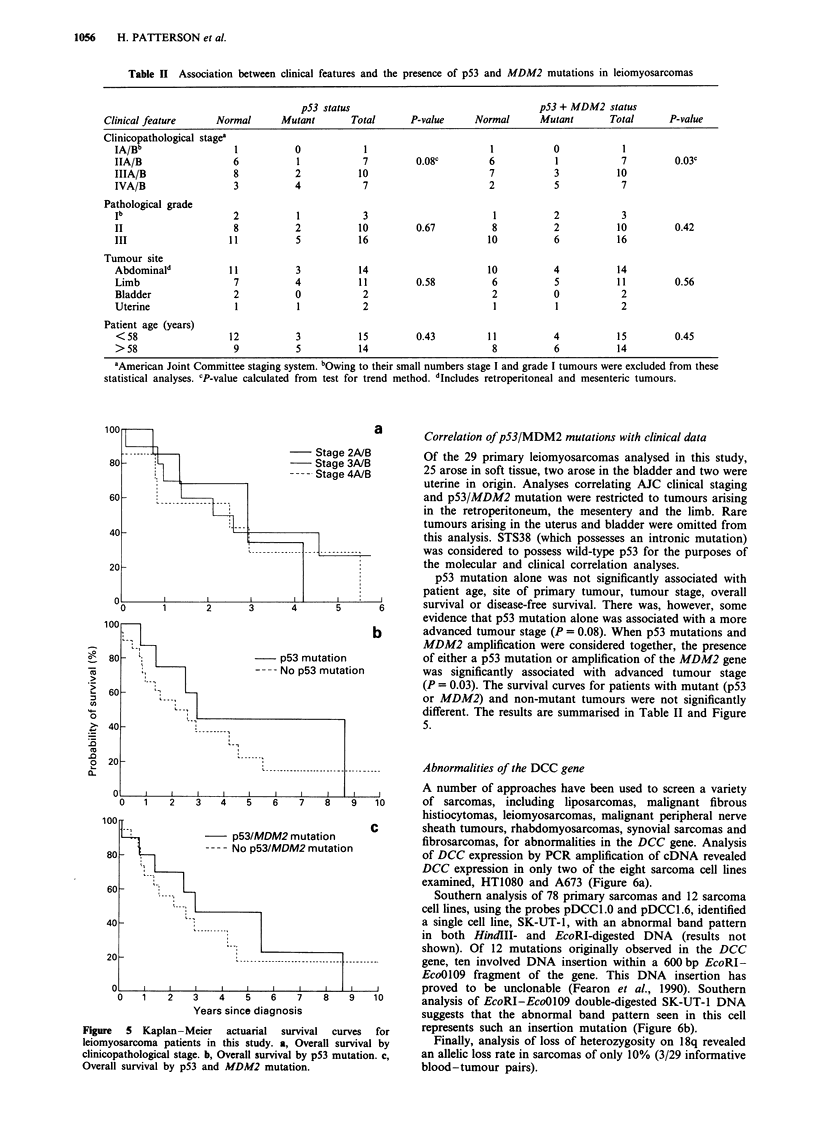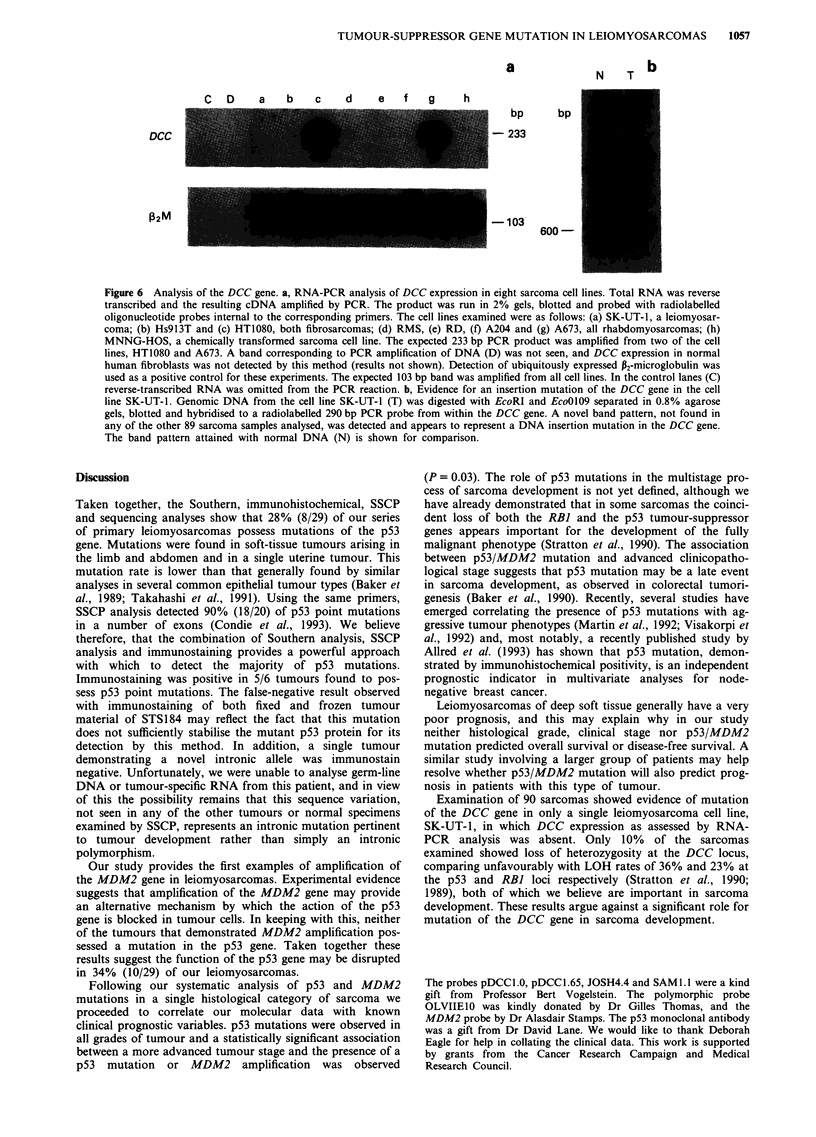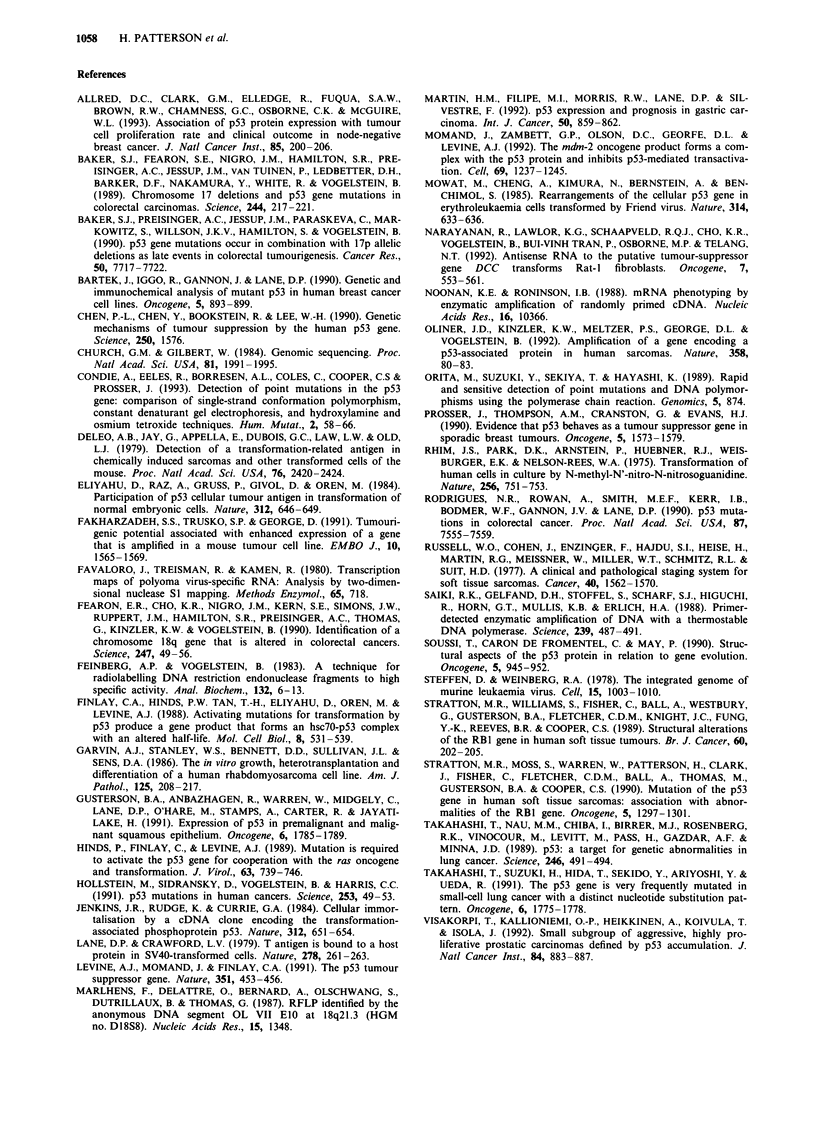# Abnormalities of the p53 MDM2 and DCC genes in human leiomyosarcomas.

**DOI:** 10.1038/bjc.1994.207

**Published:** 1994-06

**Authors:** H. Patterson, S. Gill, C. Fisher, M. G. Law, H. Jayatilake, C. D. Fletcher, M. Thomas, R. Grimer, B. A. Gusterson, C. S. Cooper

**Affiliations:** Section of Molecular Carcinogenesis, Institute of Cancer Research, Belmont, Sutton, Surrey, UK.

## Abstract

**Images:**


Br. J. Cancer (1994), 69, 1052-1058

© Macmillan Press Ltd., 1994

### Abnormalities of the p53 *MDM2* and *DCC* genes in human leiomyosarcomas

H. Patterson[1,2], S. Gill[1], C. Fisher[3], M.G. Law[4], H. Jayatilake[2], C.D.M. Fletcher[5], M. Thomas[3], R. Grimer[6], B.A. Gusterson[2] & C.S. Cooper[1]

[1]*Section of Molecular Carcinogenesis and* [2]*Section of Cell Biology and Experimental Pathology, Institute of Cancer Research, 15 Cotswold Road, Belmont, Sutton, Surrey SM2 5NG, UK;* [3]*Royal Marsden Hospital, Fulham Road, London SW3 6JJ, UK;* [4]*Section of Epidemiology, Institute of Cancer Research, 15 Cotswold Road, Belmont, Sutton, Surrey SM2 5NG, UK;* [5]*Department of Histopathology, St Thomas's Hospital, London SE1 7EH;* [6]*Royal Orthopaedic Hospital, Woodlands, Bristol Road South, Northfield, Birmingham B31 2AP, UK.*

**Summary** In this study we have screened a series of 29 primary leiomyosarcomas for abnormalities of both the p53 gene and the *MDM2* gene, which encodes a p53-associated protein. SSCP (single-strand conformation polymorphism) analysis and direct sequencing of polymerase chain reaction (PCR)-amplified DNA were used to establish that 6/29 tumours possessed point mutations of the p53 gene. Using a monoclonal antibody that recognises the p53 protein in immunohistochemical staining experiments, we observed overexpression of the p53 protein in five of the six tumours containing point mutations in the p53 gene. Southern analysis of tumour DNA revealed that 2/29 tumours demonstrated amplification of the *MDM2* gene. When considered together, these results indicate that alterations in both the p53 gene and *MDM2* gene are important in the development of a significant minority of leiomyosarcomas. In addition, we have demonstrated a significant association between the presence of abnormalities of the p53 gene or *MDM2* genes in leiomyosarcomas and a more advanced clinicopathological stage ($P = 0.03$). We have also examined the role of the *DCC* tumour-suppressor gene in the development of human soft-tissue tumours in a variety of histological types. Except for evidence of a rearrangement in a single leiomyosarcoma cell line, SK-UT-1, we have found no direct evidence to support a role for mutation of the gene in the development of human soft-tissue tumours.

Mutations of the p53 tumour-suppressor gene are the most common genetic alteration observed in human tumours including the common adult epithelial malignancies of breast (Prosser *et al.*, 1990), colon (Baker *et al.*, 1989) and lung (Takahashi *et al.*, 1989). Originally identified through its association with SV40 large T antigen in virus-transformed cells (Lane & Crawford, 1979) and as an overexpressed antigen in chemically transformed sarcoma cells (DeLeo *et al.*, 1979), p53 was initially classified as an oncogene because of its ability to transform cells in culture (Eliyahu *et al.*, 1984; Jenkins *et al.*, 1984). Subsequent analysis associated this property with a mutant p53 clone (Hinds *et al.*, 1989), and evidence of deletion of both alleles of the gene in erythroleukaemia cell lines (Mowat *et al.*, 1985), loss of heterozygosity at the p53 locus in a variety of tumours and evidence of a tumour-suppressive function for wild-type p53 (Chen *et al.*, 1990) established p53 as a tumour-suppressor gene.

The majority of p53 mutations are missense point mutations (Hollstein *et al.*, 1991; Levine *et al.*, 1991) clustered in the most highly conserved domains of the gene spanned by exons 4-9 (Soussi *et al.*, 1990). Missense mutations frequently induce changes that prolong the half-life of the p53 protein (Finlay *et al.*, 1988), and as a result mutant p53, unlike wild-type p53, accumulates in tumour cells to levels detectable by immunohistochemical methods. Elevated levels of p53 have been detected immunohistochemically in several tumour types, and when p53 has been sequenced in these tumours missense mutations are usually found (Bartek *et al.*, 1990; Rodrigues *et al.*, 1990).

The *MDM2* gene, which encodes a p53-associated protein, was first identified as a dominantly transforming oncogene in a tumorigenic mouse fibroblast cell line containing double-minute chromosomes, the cytogenic hallmark of gene amplification (Fakharzadeh *et al.*, 1991). Following this, the rat homologue of the *MDM2* gene was subsequently found to form a complex with the p53 gene, to inhibit p53-mediated transactivation (Momand *et al.*, 1992), and amplification of the *MDM2* gene has been observed in liposarcomas, malignant fibrous histiocytomas and osteosarcomas (Oliner *et al.*, 1992).

A candidate suppressor gene, *DCC* (*d*eleted in *c*olorectal *c*arcinoma), has recently been cloned by mapping a region of chromosome 18q that frequently shows allelic deletion in sporadic colorectal carcinomas (Fearon *et al.*, 1990). In keeping with the idea that *DCC* is a suppressor gene, injection of anti-sense RNA to the *DCC* gene results in the transformation of Rat-1 fibroblasts (Narayanan *et al.*, 1992).

We have previously described mutations of the p53 and *RB1* tumour-suppressor genes in human sarcomas (Stratton *et al.*, 1989, 1990). Abnormalities of the p53 gene were most frequently found in leiomyosarcomas and rhabdomyosarcomas, but only a limited series of primary leiomyosarcomas were examined and only a single exon, exon 5, of the p53 gene was screened for point mutations in these tumours. We have extended these studies by systematically screening a series of primary leiomyosarcomas for abnormalities of p53 using (i) immunohistochemical staining to screen for p53 overexpression, (ii) SSCP analysis to screen for point mutations in exons 4-9 of the p53 gene and (iii) direct sequencing analysis to define point mutations. In addition, we have also used Southern analysis to screen leiomyosarcomas for amplification of the *MDM2* gene. We subsequently used these data to determine if there was any association between abnormalities of the p53 and *MDM2* genes and patient survival or established prognostic indicators such as clinicopathological stage and histological grade for leiomyosarcomas.

We have also examined the expression and evaluated the evidence for mutation or deletion of the *DCC* gene in a large series of sarcoma cell lines and primary soft-tissue tumours of all histological types.

### Materials and methods

### *Clinical samples and cell lines*

Fresh specimens of primary soft-tissue sarcomas were obtained during surgical resection from the Royal Marsden Hospital, London and Surrey, St Thomas's Hospital, London,

Correspondence: H. Patterson, Section of Molecular Carcinogenesis, Institute of Cancer Research, 15 Cotswold Road, Belmont, Sutton, Surrey SM2 5NG, UK.

Received 6 August 1993; and in revised form 8 February 1994.

and the Royal Orthopaedic Hospital, Birmingham. Samples were immediately snap frozen in liquid nitrogen and stored at −70°C until processed. Tumours were additionally fixed in formalin or methacarn and embedded in paraffin. Non-neoplastic tissues from sarcoma patients were obtained in the form of peripheral blood lymphocytes, skeletal muscle or dermal fibroblasts. Cell lines with the exception of RMS (Garvin *et al.*, 1986) and MNNG-HOS (Rhim *et al.*, 1975) were obtained from the American Type Culture Collection (ATCC) and maintained as recommended by their supplier.

*Patient data*

Samples of leiomyosarcomas were available from 29 patients, 20 males and nine females, who underwent surgical resection between August 1980 and March 1991. Five patients underwent initial surgical resection at other hospitals and were referred to the Royal Marsden Hospital for further management. The mean age was 54 years (26–79 years). Follow-up data were available for all patients, with a median follow-up of 25 months. All patients were managed primarily with surgical resection and where appropriate received adjuvant radical radiotherapy. Isolated hepatic and pulmonary metastases were managed where possible by surgical resection. Thirteen patients received chemotherapy, for metastatic or residual disease, with adriamycin 40–60 $mg^{-2}$, ifosfamide 5 $g\ m^{-2}$ and mesna. Six patients demonstrated an objective response to chemotherapy and seven patients had progressive disease.

*Southern analysis*

DNA was extracted from tumours and normal tissue by published protocols (Steffen & Weinberg, 1978). DNA was digested with a 3- to 5-fold excess of an appropriate restriction enzyme, subjected to electrophoresis in 0.8% agarose gels and transferred to Hybond-N filters (Amersham) according to the manufacturer's instructions. Filters were hybridised according to published protocols (Church & Gilbert, 1984) to α-$^{32}$P-labelled probes prepared using random oligonucleotide primers (Feinberg & Vogelstein, 1983).

Rearrangement of the *DCC* gene was analysed using two partial cDNA probes, pDCC1.0 and pDCC1.65 (Fearon *et al.*, 1990). Amplification of the *MDM2* gene was assessed using a 1.5 kb *MDM2* probe prepared by PCR amplification of reverse-transcribed normal fibroblast RNA using the following primers: GGGAGCTCCTCGCCACCATGGTGAG-GAGCAGGCAAATG and GGGGTACCCTCATAGACA-GGTCAACTAGGGG. The PCR product was subcloned into the pBluescript vector and characterised by sequencing of its entire length. Following hybridisation to the *MDM2* probe, blots were stripped by immersion in 0.1% sodium dodecyl sulphate (SDS) at 95°C and reprobed with pDCC1.0 as a control to correct for differences between tumour samples in DNA loading and Southern transfer (Oliner *et al.*, 1992). The degree of amplification was quantified with a Joyce Loebel Chromoscan 3 scanning densitometer, using absorbance at 530 nm.

Evidence for DNA insertion within a 0.17 kb *Xba*I-*Eco*0109 fragment of the *DCC* gene (Fearon *et al.*, 1990) was examined by probing Southern blots of *Eco*RI–*Eco*0109-digested DNA of peripheral blood and tumour pairs with a probe that detects a single 600 bp *Eco*RI–*Eco*0109 fragment that spans the 0.17 kb *Xba*I–*Eco*0109 fragment. The probe was made by PCR amplification of normal DNA using the following primers: GGAGGAAGCAACTTACGGAT and TCTAGAGGGAAAATGTCATC.

Loss of heterozygosity on chromosome 18q was assessed using the following probes: pERT25 (D18S11), which detects a VNTR at 18q23, and OLVIIE10 (D18S8) (Marlhens *et al.*, 1987), SAM1.1 and JOSH4.4, which are polymorphic probes from within the *DCC* gene. pERT25 was obtained from the ATCC.

*PCR amplification*

The p53 and *DCC* genes were amplified from extracted cellular DNA by 30 cycles of the polymerase chain reaction (Saiki *et al.*, 1988) with 1 min at 92°C, 1 min at 57°C and 1 min at 72°C in a 50 μl reaction containing 200 ng of genomic DNA, 60 mM potassium chloride, 15 mM Tris–HCl pH 8.8, 2.25 mM magnesium chloride, 25 pmol of each primer, 200 μM of each dNTP and 1 unit of *Taq* polymerase (Bethesda Research Laboratories). For sequence analysis one primer of each pair was biotin labelled. For SSCP analysis one primer was end labelled with 0.3 pmol of [γ-$^{32}$P]ATP (specific activity 6,000 Ci $mmol^{-1}$) prior to the PCR reaction, or 0.6 pmol of [α-$^{32}$P]dCTP (specific activity 3,000 Ci $mmol^{-1}$) was added to each sample prior to amplification.

PCR primers for analysis of the p53 gene were as follows: lows:

Exon 4 GACCTGGTCCTCTGACTGCT and GCATTGAAGTCTCATGGAAG
Exon 5 ATCTGTTCACTTGTGCCCTG and ATCAGTGAGGAATCAGAGGC
Exon 6 GCCTCTGATTCCTCACTGAT and GGAGGGCCACTGACAACCA
Exon 7 CTTGCCACAGGTCTCCCCAA and AGGGGTCAGCGGCAAGCAGA
Exon 8 TTCCTTACTGCCTCTTGCTT and TGAATCTGAGGCATAACTGC
Exon 9 GCAGTTATGCCTCAGATTCA and ACTTTCCACTTGATAAGAGG.

*SSCP analysis*

A 2 μl aliquot of each PCR product was mixed with 8 μl loading buffer (95% formamide, 20 mM EDTA, 0.05% bromophenol blue and 0.05% xylene cyanol FF) heated to 85°C for 2 min, and 3 μl of this mixture loaded onto 6% (w/v) polyacrylamide gels (19:1, acrylamide–bisacrylamide) containing 0.5 × TBE (45 mM Tris-borate, pH 8.3, 1 mM EDTA) or 1 × TBE (90 mM Tris-borate, pH 8.3, 2 mM EDTA). Electrophoresis was performed at 40 W for 3 h at 4°C. After drying, the gel was exposed to radiographic film for 12 h to 7 days. A combination of 6% gels and 0.5 × TBE gave the best resolution of mutant bands in exons 4, 5 and 7. A combination of 6% acrylamide and 1 × TBE showed exon 8 mutations most clearly.

*Direct sequencing*

Following PCR amplification, samples were purified using streptavidin-coated magnetic beads (Dynal, UK) and sequenced as previously described (Gusterson *et al.*, 1991), using [α$^{35}$S]dATP and Sequenase Version 2.0 (United States Biochemicals).

*Analysis of gene expression using PCR*

RNA was extracted from subconfluent cultures of cell lines and normal human fibroblasts as previously described (Favaloro *et al.*, 1980). A 0.2 μg aliquot of total RNA was reverse transcribed with random hexamer primers (Noonan & Roninson, 1988) and the resulting cDNA amplified by PCR as described above. The PCR product was run in 2% (w/v) agarose gels, blotted and hybridised at 55°C to 30-mer oligonucleotide probes complementary to sequences internal to the corresponding primers, and end labelled with [γ-$^{32}$P]ATP. Filters were washed in 2 × SSC, 0.1% SDS, at 55°C and exposed to radiographic film for 30 min to 12 h. The primers were as follows:

| | |
|---|---|
| *DCC* | AGCCTCATTTTCAGCCACACA and TTCCGCCATGGTTTTTAAAT-CA |
| Internal probe | AATGGAGATGTGGTCATTCCTA-GTGATTTAT |

**Table I** Immunohistochemical and mutational analyses of the p53 gene in human leiomyosarcomas

| | *Immunohistochemistry with p53-DO7*[a] | | *SSCP analysis* | *p53 sequencing analysis* | | | |
|---|---|---|---|---|---|---|---|
| *Case* | *Paraffin* | *Frozen* | *SSCP abnormality* | *Exon* | *Codon* | *Mutation* | *Amino acid change* |
| STS16 | – | ++ | + | 5 | 151 | CCC→CAC | Pro→His |
| STS28 | +++ | ND | + | 7 | 238 | TGT→TTT | Cys→Phe |
| STS38 | – | – | + | 7 | – | Wild type[b] | |
| STS65 | – | – | ND | – | – | Homozygous deletion | |
| STS90 | – | +++ | + | 8 | 272 | GTG→ATG | Val→Met |
| STS129 | ++ | ++ | – | 5 | 158 | CGC→CAC | Arg→His |
| STS158 | – | – | ND | – | – | Homozygous deletion | |
| STS184 | – | – | + | 5 | 152 | CCG→CTG | Pro→Leu |
| STS328 | ++ | ++ | – | 6 | 216 | GTG→ATG | Val→Met |
| 20 others | – | – | – | – | – | Wild type | |

[a]The degree of immunohistochemical staining was graded as follows: (+), <10% nuclei staining; (++), 10–50% nuclei staining; (+++), >50% nuclei staining; and (–), negative results. [b]Sequencing of exon 7 in STS38 showed normal exonic sequence and splice junctions. SSCP had detected a sequence variation in intron 6. Thirty-four nucleotides upstream of exon 7 the sequence GCGCA becomes GCCCA. ND, analysis not performed.

$\beta_2$-Microglobulin ACCCCCACTGAAAAAGATGA and ATCTTCAAACCTCCATGATG
Internal probe GAACCACGTGACTTTGTCACAGCCCAAGAT.

### *Immunohistochemistry*

The mouse monoclonal antibody p53-DO7, which recognises both mutant and wild-type human p53, was used to stain both frozen and paraffin-embedded tumour material using a standard peroxidase-conjugated streptavidin–biotin method (ABC). Control slides omitting the first antibody were negative in all cases. The degree of immunostaining was graded as follows: (+), <10% nuclei staining intensely; (++), 10–50% nuclei staining intensely; (+++), >50% nuclei staining intensely.

### *Statistical analyses*

Differences in overall survival and disease-free survival between patients with or without p53/*MDM2* mutant tumours were tested using log-rank tests and were plotted according to the Kaplan–Meier method. The association between p53/*MDM2* mutation and clinical variables such as age, tumour site, American Joint Committee (AJC) clinical stage (Russell *et al.*, 1977) and histological grade were assessed using appropriate chi-square tests. The date of the definitive operative procedure was taken as the date of diagnosis.

### Results

### *Overexpression of p53 in leiomyosarcomas*

Using the monoclonal antibody p53-DO7, immunostaining demonstrated overexpression of the p53 protein in five tumours (Table I, Figure 1).

### *Abnormalities of the p53 gene detected by SSCP analysis and DNA sequencing*

SSCP was used to screen 29 leiomyosarcomas for point mutations in exons 4–9 of the p53 gene. SSCP analysis can detect point mutations in PCR-amplified segments of DNA by virtue of the fact that the rate at which PCR-amplified segments of single-stranded DNA migrate in non-denaturing gels depends upon both the strand size and base composition of the PCR product (Orita *et al.*, 1989). Abnormal SSCP band patterns were detected in five tumours (Table I, Figure 2), and three of these tumours had demonstrated p53 over-expression in immunostaining experiments.

Direct sequencing of the PCR products that gave abnormal SSCP patterns and each of the exons 4–9 in the two

**Figure 1** Immunostain analysis of p53 expression in primary leiomyosarcomas. **a**, Positive nuclear staining of a frozen section of STS90. The majority of the nuclei stain intensely. **b**, Immunostaining of a frozen section of STS90 omitting the monoclonal antibody p53-DO7. The scale bar represents 50 μm.

tumours which stained positively for the p53 protein but failed to show SSCP abnormalities revealed that in each case the DNA sequence contained a point mutation. Examples are shown in Figure 3. Six tumours possessed missense point mutations, however in STS 38 the mutation causing the SSCP abnormality occurred in intron 6, 34 bp upstream of

**Figure 2** PCR-SSCP analysis of p53 mutations in primary leiomyosarcomas. Non-denatured samples are designated D, and normal samples N. P is a positive control sample containing a mutation in exon 8. The results show (**a**) SSCP abnormalities of exon 5 in tumours STS16 and STS184, (**b**) SSCP abnormalities of exon 7 in STS28 and STS38 and (**c**) SSCP abnormality of exon 8 in STS90.

**Figure 3** p53 sequence analysis of primary leiomyosarcomas. These results show (**a**) a TGT→TTT transversion coding for the amino acid change Cys→Phe in codon 238 (exon 7) in STS28 and (**b**) a GTG→ATG transition coding for the amino acid change Val→Met in codon 272 (exon 8) in STS90.

**Figure 4** Amplification of the *MDM2* gene in STS87 and STS320. The degree of amplification seen in STS87 is maintained in each of three subsequent recurrences designated 87A, 87B, 87C. N, Normal lymphocyte DNA. The Southern blot was exposed for 96 h at $-70$°C following hybridisation to pDCC1.0, and for 16 h following hybridisation to the *MDM2* probe, to avoid overexposure of the amplified samples.

exon 7. In addition, we have previously shown that two of the tumours in this study group had undergone homozygous deletion of the p53 gene (Stratton *et al.*, 1990). Taken together, these results show that 8/29 (28%) of the leiomyosarcomas in our study group possessed missense point mutations or had undergone deletion of the p53 gene. These results are summarised in Table I.

### *Amplification of the* MDM2 *gene*

Tumours which demonstrated in excess of a 5-fold increase in the signal with the *MDM2* probe relative to the signal seen with pDCC1.0 were considered to show *MDM2* amplification. pDCC1.0 was considered a reasonable control probe as hemizygous loss of chromosome 18q, the *DCC* locus, as reported in this paper, occurs in only 10% of sarcomas, a level consistent with the background loss observed with most genetic markers. Southern analysis demonstrated amplification of the *MDM2* gene in 2/29 leiomyosarcomas. STS 87, a metastasis from a primary tumour, showed a 60-fold amplification in the *MDM2* gene. The amplification was maintained at a similar level in each of three subsequent recurrences. A 120-fold amplification of the *MDM2* gene was seen in STS 320, a locally advanced primary tumour (Figure 4). Although densitometry may be unable to measure amplification of this degree accurately, the levels of *MDM2* amplification in these tumours are clearly significant.

**Table II** Association between clinical features and the presence of p53 and *MDM2* mutations in leiomyosarcomas

| *Clinical feature* | *p53 status* Normal | Mutant | Total | *P-value* | *p53 + MDM2 status* Normal | Mutant | Total | *P-value* |
|---|---|---|---|---|---|---|---|---|
| Clinicopathological stage[a] | | | | | | | | |
| IA/B[b] | 1 | 0 | 1 | | 1 | 0 | 1 | |
| IIA/B | 6 | 1 | 7 | 0.08[c] | 6 | 1 | 7 | 0.03[c] |
| IIIA/B | 8 | 2 | 10 | | 7 | 3 | 10 | |
| IVA/B | 3 | 4 | 7 | | 2 | 5 | 7 | |
| Pathological grade | | | | | | | | |
| I[b] | 2 | 1 | 3 | | 1 | 2 | 3 | |
| II | 8 | 2 | 10 | 0.67 | 8 | 2 | 10 | 0.42 |
| III | 11 | 5 | 16 | | 10 | 6 | 16 | |
| Tumour site | | | | | | | | |
| Abdominal[d] | 11 | 3 | 14 | | 10 | 4 | 14 | |
| Limb | 7 | 4 | 11 | 0.58 | 6 | 5 | 11 | 0.56 |
| Bladder | 2 | 0 | 2 | | 2 | 0 | 2 | |
| Uterine | 1 | 1 | 2 | | 1 | 1 | 2 | |
| Patient age (years) | | | | | | | | |
| <58 | 12 | 3 | 15 | 0.43 | 11 | 4 | 15 | 0.45 |
| >58 | 9 | 5 | 14 | | 8 | 6 | 14 | |

[a]American Joint Committee staging system. [b]Owing to their small numbers stage I and grade I tumours were excluded from these statistical analyses. [c]*P*-value calculated from test for trend method. [d]Includes retroperitoneal and mesenteric tumours.

**Figure 5** Kaplan–Meier actuarial survival curves for leiomyosarcoma patients in this study. **a**, Overall survival by clinicopathological stage. **b**, Overall survival by p53 mutation. **c**, Overall survival by p53 and *MDM2* mutation.

### *Correlation of p53/*MDM2 *mutations with clinical data*

Of the 29 primary leiomyosarcomas analysed in this study, 25 arose in soft tissue, two arose in the bladder and two were uterine in origin. Analyses correlating AJC clinical staging and p53/*MDM2* mutation were restricted to tumours arising in the retroperitoneum, the mesentery and the limb. Rare tumours arising in the uterus and bladder were omitted from this analysis. STS38 (which possesses an intronic mutation) was considered to possess wild-type p53 for the purposes of the molecular and clinical correlation analyses.

p53 mutation alone was not significantly associated with patient age, site of primary tumour, tumour stage, overall survival or disease-free survival. There was, however, some evidence that p53 mutation alone was associated with a more advanced tumour stage ($P = 0.08$). When p53 mutations and *MDM2* amplification were considered together, the presence of either a p53 mutation or amplification of the *MDM2* gene was significantly associated with advanced tumour stage ($P = 0.03$). The survival curves for patients with mutant (p53 or *MDM2*) and non-mutant tumours were not significantly different. The results are summarised in Table II and Figure 5.

### *Abnormalities of the* DCC *gene*

A number of approaches have been used to screen a variety of sarcomas, including liposarcomas, malignant fibrous histiocytomas, leiomyosarcomas, malignant peripheral nerve sheath tumours, rhabdomyosarcomas, synovial sarcomas and fibrosarcomas, for abnormalities in the *DCC* gene. Analysis of *DCC* expression by PCR amplification of cDNA revealed *DCC* expression in only two of the eight sarcoma cell lines examined, HT1080 and A673 (Figure 6a).

Southern analysis of 78 primary sarcomas and 12 sarcoma cell lines, using the probes pDCC1.0 and pDCC1.6, identified a single cell line, SK-UT-1, with an abnormal band pattern in both *Hind*III- and *Eco*RI-digested DNA (results not shown). Of 12 mutations originally observed in the *DCC* gene, ten involved DNA insertion within a 600 bp *Eco*RI–*Eco*0109 fragment of the gene. This DNA insertion has proved to be unclonable (Fearon *et al.*, 1990). Southern analysis of *Eco*RI–*Eco*0109 double-digested SK-UT-1 DNA suggests that the abnormal band pattern seen in this cell represents such an insertion mutation (Figure 6b).

Finally, analysis of loss of heterozygosity on 18q revealed an allelic loss rate in sarcomas of only 10% (3/29 informative blood–tumour pairs).

**Figure 6** Analysis of the *DCC* gene. **a**, RNA-PCR analysis of *DCC* expression in eight sarcoma cell lines. Total RNA was reverse transcribed and the resulting cDNA amplified by PCR. The product was run in 2% gels, blotted and probed with radiolabelled oligonucleotide probes internal to the corresponding primers. The cell lines examined were as follows: (a) SK-UT-1, a leiomyosarcoma; (b) Hs913T and (c) HT1080, both fibrosarcomas; (d) RMS, (e) RD, (f) A204 and (g) A673, all rhabdomyosarcomas; (h) MNNG-HOS, a chemically transformed sarcoma cell line. The expected 233 bp PCR product was amplified from two of the cell lines, HT1080 and A673. A band corresponding to PCR amplification of DNA (D) was not seen, and *DCC* expression in normal human fibroblasts was not detected by this method (results not shown). Detection of ubiquitously expressed $\beta_2$-microglobulin was used as a positive control for these experiments. The expected 103 bp band was amplified from all cell lines. In the control lanes (C) reverse-transcribed RNA was omitted from the PCR reaction. **b**, Evidence for an insertion mutation of the *DCC* gene in the cell line SK-UT-1. Genomic DNA from the cell line SK-UT-1 (T) was digested with *Eco*RI and *Eco*0109 separated in 0.8% agarose gels, blotted and hybridised to a radiolabelled 290 bp PCR probe from within the *DCC* gene. A novel band pattern, not found in any of the other 89 sarcoma samples analysed, was detected and appears to represent a DNA insertion mutation in the *DCC* gene. The band pattern attained with normal DNA (N) is shown for comparison.

### Discussion

Taken together, the Southern, immunohistochemical, SSCP and sequencing analyses show that 28% (8/29) of our series of primary leiomyosarcomas possess mutations of the p53 gene. Mutations were found in soft-tissue tumours arising in the limb and abdomen and in a single uterine tumour. This mutation rate is lower than that generally found by similar analyses in several common epithelial tumour types (Baker *et al.*, 1989; Takahashi *et al.*, 1991). Using the same primers, SSCP analysis detected 90% (18/20) of p53 point mutations in a number of exons (Condie *et al.*, 1993). We believe therefore, that the combination of Southern analysis, SSCP analysis and immunostaining provides a powerful approach with which to detect the majority of p53 mutations. Immunostaining was positive in 5/6 tumours found to possess p53 point mutations. The false-negative result observed with immunostaining of both fixed and frozen tumour material of STS184 may reflect the fact that this mutation does not sufficiently stabilise the mutant p53 protein for its detection by this method. In addition, a single tumour demonstrating a novel intronic allele was immunostain negative. Unfortunately, we were unable to analyse germ-line DNA or tumour-specific RNA from this patient, and in view of this the possibility remains that this sequence variation, not seen in any of the other tumours or normal specimens examined by SSCP, represents an intronic mutation pertinent to tumour development rather than simply an intronic polymorphism.

Our study provides the first examples of amplification of the *MDM2* gene in leiomyosarcomas. Experimental evidence suggests that amplification of the *MDM2* gene may provide an alternative mechanism by which the action of the p53 gene is blocked in tumour cells. In keeping with this, neither of the tumours that demonstrated *MDM2* amplification possessed a mutation in the p53 gene. Taken together these results suggest the function of the p53 gene may be disrupted in 34% (10/29) of our leiomyosarcomas.

Following our systematic analysis of p53 and *MDM2* mutations in a single histological category of sarcoma we proceeded to correlate our molecular data with known clinical prognostic variables. p53 mutations were observed in all grades of tumour and a statistically significant association between a more advanced tumour stage and the presence of a p53 mutation or *MDM2* amplification was observed ($P = 0.03$). The role of p53 mutations in the multistage process of sarcoma development is not yet defined, although we have already demonstrated that in some sarcomas the coincident loss of both the *RB1* and the p53 tumour-suppressor genes appears important for the development of the fully malignant phenotype (Stratton *et al.*, 1990). The association between p53/*MDM2* mutation and advanced clinicopathological stage suggests that p53 mutation may be a late event in sarcoma development, as observed in colorectal tumorigenesis (Baker *et al.*, 1990). Recently, several studies have emerged correlating the presence of p53 mutations with aggressive tumour phenotypes (Martin *et al.*, 1992; Visakorpi *et al.*, 1992) and, most notably, a recently published study by Allred *et al.* (1993) has shown that p53 mutation, demonstrated by immunohistochemical positivity, is an independent prognostic indicator in multivariate analyses for node-negative breast cancer.

Leiomyosarcomas of deep soft tissue generally have a very poor prognosis, and this may explain why in our study neither histological grade, clinical stage nor p53/*MDM2* mutation predicted overall survival or disease-free survival. A similar study involving a larger group of patients may help resolve whether p53/*MDM2* mutation will also predict prognosis in patients with this type of tumour.

Examination of 90 sarcomas showed evidence of mutation of the *DCC* gene in only a single leiomyosarcoma cell line, SK-UT-1, in which *DCC* expression as assessed by RNA-PCR analysis was absent. Only 10% of the sarcomas examined showed loss of heterozygosity at the *DCC* locus, comparing unfavourably with LOH rates of 36% and 23% at the p53 and *RB1* loci respectively (Stratton *et al.*, 1990; 1989), both of which we believe are important in sarcoma development. These results argue against a significant role for mutation of the *DCC* gene in sarcoma development.

The probes pDCC1.0, pDCC1.65, JOSH4.4 and SAM1.1 were a kind gift from Professor Bert Vogelstein. The polymorphic probe OLVIIE10 was kindly donated by Dr Gilles Thomas, and the *MDM2* probe by Dr Alasdair Stamps. The p53 monoclonal antibody was a gift from Dr David Lane. We would like to thank Deborah Eagle for help in collating the clinical data. This work is supported by grants from the Cancer Research Campaign and Medical Research Council.

### References

ALLRED, D.C., CLARK, G.M., ELLEDGE, R., FUQUA, S.A.W., BROWN, R.W., CHAMNESS, G.C., OSBORNE, C.K. & MCGUIRE, W.L. (1993). Association of p53 protein expression with tumour cell proliferation rate and clinical outcome in node-negative breast cancer. *J. Natl Cancer Inst.*, **85**, 200-206.

BAKER, S.J., FEARON, S.E., NIGRO, J.M., HAMILTON, S.R., PREISINGER, A.C., JESSUP, J.M., VAN TUINEN, P., LEDBETTER, D.H., BARKER, D.F., NAKAMURA, Y., WHITE, R. & VOGELSTEIN, B. (1989). Chromosome 17 deletions and p53 gene mutations in colorectal carcinomas. *Science*, **244**, 217-221.

BAKER, S.J., PREISINGER, A.C., JESSUP, J.M., PARASKEVA, C., MARKOWITZ, S., WILLSON, J.K.V., HAMILTON, S. & VOGELSTEIN, B. (1990). p53 gene mutations occur in combination with 17p allelic deletions as late events in colorectal tumourigenesis. *Cancer Res.*, **50**, 7717-7722.

BARTEK, J., IGGO, R., GANNON, J. & LANE, D.P. (1990). Genetic and immunochemical analysis of mutant p53 in human breast cancer cell lines. *Oncogene*, **5**, 893-899.

CHEN, P.-L., CHEN, Y., BOOKSTEIN, R. & LEE, W.-H. (1990). Genetic mechanisms of tumour suppression by the human p53 gene. *Science*, **250**, 1576.

CHURCH, G.M. & GILBERT, W. (1984). Genomic sequencing. *Proc. Natl Acad. Sci. USA*, **81**, 1991-1995.

CONDIE, A., EELES, R., BORRESEN, A.L., COLES, C., COOPER, C.S & PROSSER, J. (1993). Detection of point mutations in the p53 gene: comparison of single-strand conformation polymorphism, constant denaturant gel electrophoresis, and hydroxylamine and osmium tetroxide techniques. *Hum. Mutat.*, **2**, 58-66.

DELEO, A.B., JAY, G., APPELLA, E., DUBOIS, G.C., LAW, L.W. & OLD, L.J. (1979). Detection of a transformation-related antigen in chemically induced sarcomas and other transformed cells of the mouse. *Proc. Natl Acad. Sci. USA*, **76**, 2420-2424.

ELIYAHU, D., RAZ, A., GRUSS, P., GIVOL, D. & OREN, M. (1984). Participation of p53 cellular tumour antigen in transformation of normal embryonic cells. *Nature*, **312**, 646-649.

FAKHARZADEH, S.S., TRUSKO, S.P. & GEORGE, D. (1991). Tumourigenic potential associated with enhanced expression of a gene that is amplified in a mouse tumour cell line. *EMBO J.*, **10**, 1565-1569.

FAVALORO, J., TREISMAN, R. & KAMEN, R. (1980). Transcription maps of polyoma virus-specific RNA: Analysis by two-dimensional nuclease S1 mapping. *Methods Enzymol.*, **65**, 718.

FEARON, E.R., CHO, K.R., NIGRO, J.M., KERN, S.E., SIMONS, J.W., RUPPERT, J.M., HAMILTON, S.R., PREISINGER, A.C., THOMAS, G., KINZLER, K.W. & VOGELSTEIN, B. (1990). Identification of a chromosome 18q gene that is altered in colorectal cancers. *Science*, **247**, 49-56.

FEINBERG, A.P. & VOGELSTEIN, B. (1983). A technique for radiolabelling DNA restriction endonuclease fragments to high specific activity. *Anal. Biochem.*, **132**, 6-13.

FINLAY, C.A., HINDS, P.W. TAN, T.-H., ELIYAHU, D., OREN, M. & LEVINE, A.J. (1988). Activating mutations for transformation by p53 produce a gene product that forms an hsc70-p53 complex with an altered half-life. *Mol. Cell Biol.*, **8**, 531-539.

GARVIN, A.J., STANLEY, W.S., BENNETT, D.D., SULLIVAN, J.L. & SENS, D.A. (1986). The *in vitro* growth, heterotransplantation and differentiation of a human rhabdomyosarcoma cell line. *Am. J. Pathol.*, **125**, 208-217.

GUSTERSON, B.A., ANBAZHAGEN, R., WARREN, W., MIDGELY, C., LANE, D.P., O'HARE, M., STAMPS, A., CARTER, R. & JAYATILAKE, H. (1991). Expression of p53 in premalignant and malignant squamous epithelium. *Oncogene*, **6**, 1785-1789.

HINDS, P., FINLAY, C., & LEVINE, A.J. (1989). Mutation is required to activate the p53 gene for cooperation with the *ras* oncogene and transformation. *J. Virol.*, **63**, 739-746.

HOLLSTEIN, M., SIDRANSKY, D., VOGELSTEIN, B. & HARRIS, C.C. (1991). p53 mutations in human cancers. *Science*, **253**, 49-53.

JENKINS, J.R., RUDGE, K. & CURRIE, G.A. (1984). Cellular immortalisation by a cDNA clone encoding the transformation-associated phosphoprotein p53. *Nature*, **312**, 651-654.

LANE, D.P. & CRAWFORD, L.V. (1979). T antigen is bound to a host protein in SV40-transformed cells. *Nature*, **278**, 261-263.

LEVINE, A.J., MOMAND, J. & FINLAY, C.A. (1991). The p53 tumour suppressor gene. *Nature*, **351**, 453-456.

MARLHENS, F., DELATTRE, O., BERNARD, A., OLSCHWANG, S., DUTRILLAUX, B. & THOMAS, G. (1987). RFLP identified by the anonymous DNA segment OL VII E10 at 18q21.3 (HGM no. D18S8). *Nucleic Acids Res.*, **15**, 1348.

MARTIN, H.M., FILIPE, M.I., MORRIS, R.W., LANE, D.P. & SILVESTRE, F. (1992). p53 expression and prognosis in gastric carcinoma. *Int. J. Cancer*, **50**, 859-862.

MOMAND, J., ZAMBETT, G.P., OLSON, D.C., GEORFE, D.L. & LEVINE, A.J. (1992). The *mdm*-2 oncogene product forms a complex with the p53 protein and inhibits p53-mediated transactivation. *Cell*, **69**, 1237-1245.

MOWAT, M., CHENG, A., KIMURA, N., BERNSTEIN, A. & BENCHIMOL, S. (1985). Rearrangements of the cellular p53 gene in erythroleukaemia cells transformed by Friend virus. *Nature*, **314**, 633-636.

NARAYANAN, R., LAWLOR, K.G., SCHAAPVELD, R.Q.J., CHO, K.R., VOGELSTEIN, B., BUI-VINH TRAN, P., OSBORNE, M.P. & TELANG, N.T. (1992). Antisense RNA to the putative tumour-suppressor gene *DCC* transforms Rat-1 fibroblasts. *Oncogene*, **7**, 553-561.

NOONAN, K.E. & RONINSON, I.B. (1988). mRNA phenotyping by enzymatic amplification of randomly primed cDNA. *Nucleic Acids Res.*, **16**, 10366.

OLINER, J.D., KINZLER, K.W., MELTZER, P.S., GEORGE, D.L. & VOGELSTEIN, B. (1992). Amplification of a gene encoding a p53-associated protein in human sarcomas. *Nature*, **358**, 80-83.

ORITA, M., SUZUKI, Y., SEKIYA, T. & HAYASHI, K. (1989). Rapid and sensitive detection of point mutations and DNA polymorphisms using the polymerase chain reaction. *Genomics*, **5**, 874.

PROSSER, J., THOMPSON, A.M., CRANSTON, G. & EVANS, H.J. (1990). Evidence that p53 behaves as a tumour suppressor gene in sporadic breast tumours. *Oncogene*, **5**, 1573-1579.

RHIM, J.S., PARK, D.K., ARNSTEIN, P., HUEBNER, R.J., WEISBURGER, E.K. & NELSON-REES, W.A. (1975). Transformation of human cells in culture by N-methyl-N'-nitro-N-nitrosoguanidine. *Nature*, **256**, 751-753.

RODRIGUES, N.R., ROWAN, A., SMITH, M.E.F., KERR, I.B., BODMER, W.F., GANNON, J.V. & LANE, D.P. (1990). p53 mutations in colorectal cancer. *Proc. Natl Acad. Sci. USA*, **87**, 7555-7559.

RUSSELL, W.O., COHEN, J., ENZINGER, F., HAJDU, S.I., HEISE, H., MARTIN, R.G., MEISSNER, W., MILLER, W.T., SCHMITZ, R.L. & SUIT, H.D. (1977). A clinical and pathological staging system for soft tissue sarcomas. *Cancer*, **40**, 1562-1570.

SAIKI, R.K., GELFAND, D.H., STOFFEL, S., SCHARF, S.J., HIGUCHI, R., HORN, G.T., MULLIS, K.B. & ERLICH, H.A. (1988). Primer-detected enzymatic amplification of DNA with a thermostable DNA polymerase. *Science*, **239**, 487-491.

SOUSSI, T., CARON DE FROMENTEL, C. & MAY, P. (1990). Structural aspects of the p53 protein in relation to gene evolution. *Oncogene*, **5**, 945-952.

STEFFEN, D. & WEINBERG, R.A. (1978). The integrated genome of murine leukaemia virus. *Cell*, **15**, 1003-1010.

STRATTON, M.R., WILLIAMS, S., FISHER, C., BALL, A., WESTBURY, G., GUSTERSON, B.A., FLETCHER, C.D.M., KNIGHT, J.C., FUNG, Y.-K., REEVES, B.R. & COOPER, C.S. (1989). Structural alterations of the RB1 gene in human soft tissue tumours. *Br. J. Cancer*, **60**, 202-205.

STRATTON, M.R., MOSS, S., WARREN, W., PATTERSON, H., CLARK, J., FISHER, C., FLETCHER, C.D.M., BALL, A., THOMAS, M., GUSTERSON, B.A. & COOPER, C.S. (1990). Mutation of the p53 gene in human soft tissue sarcomas: association with abnormalities of the RB1 gene. *Oncogene*, **5**, 1297-1301.

TAKAHASHI, T., NAU, M.M., CHIBA, I., BIRRER, M.J., ROSENBERG, R.K., VINOCOUR, M., LEVITT, M., PASS, H., GAZDAR, A.F. & MINNA, J.D. (1989). p53: a target for genetic abnormalities in lung cancer. *Science*, **246**, 491-494.

TAKAHASHI, T., SUZUKI, H., HIDA, T., SEKIDO, Y., ARIYOSHI, Y. & UEDA, R. (1991). The p53 gene is very frequently mutated in small-cell lung cancer with a distinct nucleotide substitution pattern. *Oncogene*, **6**, 1775-1778.

VISAKORPI, T., KALLIONIEMI, O.-P., HEIKKINEN, A., KOIVULA, T. & ISOLA, J. (1992). Small subgroup of aggressive, highly proliferative prostatic carcinomas defined by p53 accumulation. *J. Natl Cancer Inst.*, **84**, 883-887.